# One-Step Homology Mediated CRISPR-Cas Editing in Zygotes for Generating Genome Edited Cattle

**DOI:** 10.1089/crispr.2020.0047

**Published:** 2020-12-17

**Authors:** Ki-Eun Park, Juli Foster Frey, Jerel Waters, Sean G. Simpson, Chris Coutu, Sarah Plummer, Matthew Campbell, David M. Donovan, Bhanu P. Telugu

**Affiliations:** ^1^ Animal and Avian Sciences, University of Maryland, College Park, Maryland, USA; ^2^ RenOVAte Biosciences, Inc., Reisterstown, Maryland, USA; ^3^ Animal Biosciences and Biotechnology Laboratory, USDA, ARS, Beltsville, Maryland, USA; ^4^ Thomas D. Morris Inc., Reisterstown, Maryland, USA; ^5^ Genus plc, Madison, Wisconsin, USA.

## Abstract

Selective breeding and genetic modification have been the cornerstone of animal agriculture. However, the current strategy of breeding animals over multiple generations to introgress novel alleles is not practical in addressing global challenges such as climate change, pandemics, and the predicted need to feed a population of 9 billion by 2050. Consequently, genome editing in zygotes to allow for seamless introgression of novel alleles is required, especially in cattle with long generation intervals. We report for the first time the use of CRISPR-Cas genome editors to introduce novel *PRNP* allelic variants that have been shown to provide resilience towards human prion pandemics. From one round of embryo injections, we have established six pregnancies and birth of seven edited offspring, with two founders showing >90% targeted homology-directed repair modifications. This study lays out the framework for *in vitro* optimization, unbiased deep-sequencing to identify editing outcomes, and generation of high frequency homology-directed repair–edited calves.

## Introduction

Genetic modification of livestock has a long and storied history, beginning with the domestication of animals. Initially, animals exhibiting traits favorable for domestication and performance were chosen for breeding (mass selection). Then with time, the process became more sophisticated and included pedigree selection, marker-assisted selection, and with the sequencing of genomes, genome-wide selection. Transgenics marked the first major milestone that allowed for introgression of novel alleles and traits into livestock animals.^1^ This in combination with somatic cell nuclear transfer (SCNT)^2,3^ became the main staple for generating genetically modified livestock. The recent discovery and successful validation of genome editors (ZFNs, TALENs, and CRISPR-Cas9) will now allow for genetic modification directly in the zygote bypassing the need for SCNT.^4–10^ Among the genome editors, CRISPR-Cas9 has been widely employed for livestock genome editing.^8–10^ The system uses a 17–20 nucleotide RNA sequence (“spacer”) as a guide along with a universal sequence (“tracr”) that permits loading into the Cas9 protein (single guide RNA [sgRNA]).^11–13^ When a precomplexed Cas9 protein and sgRNA ribonucleoprotein (RNP) complex is delivered into the target cell or embryo, the Cas9 introduces double-stranded breaks at target DNA sites.^13^ At a lower frequency, the double-stranded breaks can undergo homology-directed repair (HDR) in the presence of homologous repair template, or at a higher frequency by error-prone nonhomologous end-joining (NHEJ) pathways^14^ Several recent manuscripts highlighted the feasibility of editing bovine genome in somatic cells,^15–20^ and direct delivery of editors into zygotes^6,21–23^ However, no manuscript to date has reported the generation of live calves with novel variants by CRISPR-Cas-mediated HDR editing of bovine genome directly in zygotes. The main goal of this study was to establish a pipeline for (1) rational design and selection of targeting reagents, (2) high throughput screening approaches to evaluate editing outcomes, (3) optimized techniques for embryo injections to maximize HDR outcomes, and (4) ultimately, generation of genome-edited calves with HDR mediated introgression of novel variants at high efficiency. As a proof of concept, introduction of novel allelic *PRNP* (prion protein) variants has been chosen for validation of the optimization pipeline and for the generation of cattle resistant to prion diseases.

Misfolded cellular prion proteins (PrP) result in degenerative central nervous system disorders referred to as transmissible spongiform encephalopathies,^24^ such as bovine spongiform encephalopathy (BSE) in cattle, scrapie in sheep, and Creutzfeldt-Jakob disease in humans.^24^ Even though the ban of ruminant derived feed supplements helped reduce the incidence of BSE cases, the risk of atypical BSE resulting from spontaneous misfolding of endogenous PrP remains a concern.^25–27^ To address this concern, *PRNP* null cattle have been generated via SCNT.^28^ With the availability of CRISPRs, several groups are revisiting generating *PRNP* null cattle, although, most attempts have been limited to efforts in vitro.^17,18^ Several natural variants of *PRNP* have been identified that provided protection against inherited prion diseases.^29,30^ One particular PrP variant with glycine (G) at position 127 replaced with valine (V), referred to as G127V, was demonstrated to provide dominant-negative protection against the disease (in heterozygosity) in the “Fore” population of Papua New Guinea during the Kuru prion epidemic.^31^ In an elegant study, mice null for endogenous *Prnp* and transgenic for human G127V variant conferred resistance against all 18 prion disease isolates.^31^ This single amino acid substitution (G127V) was as protective as deletion or knockout of the protein.^31^ Even though mouse has been used as a surrogate in this and other studies,^31–36^ replicating these findings in cattle—a natural host to the disease—will be beneficial in both confirming these findings and generating disease-resistant elite cattle breeds. In this study, as a proof of concept, our aim was to engineer G127V allele in cattle using CRISPR-Cas genome editors.

## Materials and Methods

### Reagents

All chemicals were obtained from Sigma-Aldrich Company (St. Louis, MO) unless stated otherwise. All CRISPR-Cas reagents, targeting oligos, and PCR primers used in this study were purchased from IDT DNA Technologies (Coralville, IA) and are shown in Table 1.

**Table 1.** **Sequence of nucleotide reagents used in the study**

Category	Sequence
F guide sgRNA spacer sequence	GCAGUGGUAGGGGGCCUUGG
R guide sgRNA spacer sequence	UUCCCAGCAUGUAGCCACCA
PCR primer F (Fig.1)	GAGCCGATACCCAGGACAGG
PCR primer R (Fig.1)	GTCAGTTTCGGTGAAGTTCTC (Product = 526 bp; Acc I digest yields 301 + 225 bp)
PCR primer F1 (Figs. 2–4)	ACCTGGAGGAGGATGGAACA
PCR primer R2 (Figs.2–4)	CACTTGCCCCTCGTTGGTAA (Product = 641 bp; Acc I digest yields 328 + 313 bp)
MiSeq initial PCR For	GGTTCTCTTTGTGGCCATGTGG
MiSeq initial PCR Rev	AAGGAACACACAGTCACCACCA
MiSeq Step 1 PCR For	ACACTCTTTCCCTACACGACGCTCTTCCGATCTGGTACCCACGGTCAATGGAACA
MiSeq Step 1 PCR Rev	GTGACTGGAGTTCAGACGTGTGCTCTTCCGATCTTTGGCAGTGACTATGAGGACCG
iSeq F	AGGTGGTACCCACGGTCAAT
iSeq R	CATGCACAAAGTTGTTCTGGTT (Product = 257 bp)
Sense symmetrical oligo	GGTACCCACGGTCAATGGAACAAACCCAGTAAGCCAAAAACCAACATGAAGCATGTGGCAGGAGCTGCTGCAGCTGGAGCAGTGGTAGGGGGCCTTGGTGTATACATGCTGGGAAGTGCCATGAGCAGGCCTCTTATACATTTTGGCAGTGACTATGAGGACCGTTACTATCGTGAAAACATGCACCGTTACCCCAACCA
Antisense symmetrical oligo	TGGTTGGGGTAACGGTGCATGTTTTCACGATAGTAACGGTCCTCATAGTCACTGCCAAAATGTATAAGAGGCCTGCTCATGGCACTTCCCAGCATGTATACACCAAGGCCCCCTACCACTGCTCCAGCTGCAGCAGCTCCTGCCACATGCTTCATGTTGGTTTTTGGCTTACTGGGTTTGTTCCATTGACCGTGGGTACC
Antisense asymmetrical oligo	GGCCTGCTCATGGCACTTCCCAGCATGTATACACCAAGGCCCCCTACCACTGCTCCAGCTGCAGCAGCTCCTGCCACATGCTTCATGTTGGTTTTTGGCTTACTGGGTTTGTTCCATTGACCGTGGG
Reverse asymmetrical oligo	GGGTAACGGTGCATGTTTTCACGATAGTAACGGTCCTCATAGTCACTGCCAAAATGTATAAGAGGCCTGCTCATGGCACTTCCCAGCATGTATACACCAAGGCCCCCTACCACTGCTCCAGCTGCAG

F, forward; oligo, oligodeoxynucleotide; R, reverse; sgRNA, single guide RNA.

### Animal experimental assurance

All experiments involving live animals were performed in accordance with the approved guidelines of the University of Maryland and Thomas D. Morris Inc. institutional animal care and use committees. All experimental protocols involving live animals were approved by the institutional animal care and use committees at both Institutes (UMD protocol No. 1418824-1 and TDMI protocol No. 18-005: Genome editing in Cattle).

### In vitro maturation, in vitro fertilization, and microinjection

Cumulus oocyte complexes (COCs) from abattoir cattle were purchased from ART Inc. (Madison, WI) or DeSoto Biosciences Inc. (Seymour, TN) and shipped to the lab overnight in Maturation medium at 38.5°C. Twenty-two hours (h) after being placed in the maturation medium, in vitro fertilization (IVF) was performed in accordance to an established protocol using frozen semen.^37^ Briefly, two straws of thawed semen were mixed with Dulbecco's phosphate buffered saline (DPBS) containing 1 mg/mL BSA to a final volume of 10 mL and centrifuged at 1000 *g*, 25°C for 4 minutes, followed by two washes of spermatozoa in DPBS. After the final wash, spermatozoa were co-incubated with matured COCs for 6 h in four-well NUNC dishes (50 COCs/500 μL) in modified Brackett and Oliphant isotonic medium containing 3 mg/mL fatty-acid-free BSA and supplemented with PHE (20 μM D-penicillamine, 10 μM hypotaurine, and 1 μM epinephrine),^38^ at a final concentration of 2 × 10^6^ spermatozoa/mL at 38.5°C and 5% CO_2_. Six hours after IVF, the presumptive zygotes were vortexed in 0.1% hyaluronidase in HEPES-buffered medium containing 0.01% poly vinyl alcohol (PVA) for 4 minutes to remove the cumulus cells and extraneous spermatozoa. Presumptive zygotes were microinjected with a mixture of commercially sourced Cas9 RNP (Cas9 protein and sgRNA) and single-stranded DNA using a FemtoJet microinjector (Eppendorf, Germany). The microinjected embryos were cultured to blastocyst stage in serum-free medium (BO-IVC; IVF Bioscience, UK) for 8 days at 38.5°C, 5% CO_2_, 5% O_2_, and 100% humidity. Progression to cleavage and blastocyst stage of embryo development rate was recorded on days 2 and 8 post IVF respectively.

### Optimization of CRISPR-Cas targeting reagents

#### Experiment 1. Design and validation of CRISPR-Cas sgRNA

**FIG. 1.**
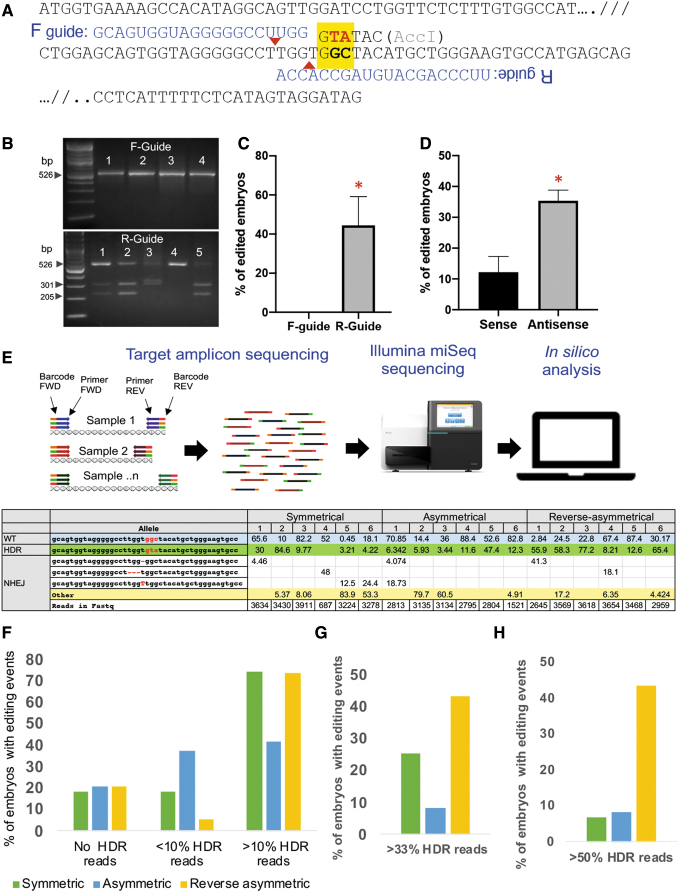
Identifying optimal CRISPR-Cas targeting reagents. **(A)** Schematic outlining the target site of the endogenous bovine *PRNP* locus. The coding sequence was truncated for convenience (indicated by “//”). The site coding for glycine (GGC) at amino acid position 127 targeted for conversion to valine (GTA) is highlighted in yellow. Successful gene targeting will result in the generation of AccI restriction enzyme site (*GTATAC*). Two CRISPR spacer sequences targeting the sense strand (F-guide) and antisense strand (R-guide) are shown above the target site. Putative cut-site is shown as a red triangle for each guide. **(B)** Representative agarose gel electrophoresis image indicating that AccI site introduction into genomic DNA results only from R-guide, and not from F-guide. Targeted genomic region was PCR amplified from embryos (numbered), amplicon fragment gel purified, AccI digested, and digestion fragments resolved on a 2% agarose gel. **(C)** Results from gene targeting with F- and R-guide from three triplicate experiments. No successful gene targeting was identified with F- guide (*n* = 14 blastocysts), whereas R-guide resulted in successful targeting with a statistically significant finding (*n* = 16 blastocysts; *P < 0.05). **(D)** Results from gene targeting with R-guide and oligos targeting either sense (*n* = 39 blastocysts) or antisense strand (*n* = 39 blastocysts). Antisense oligo resulted in better targeting efficiencies from duplicate experiments (*P < 0.05). **(E)** Schematic outlining Illumina miSeq targeting amplicon sequencing of blastocysts from gene targeting of embryos with various iterations of antisense oligos (sense, antisense, reverse-asymmetric). FastQ files from the miSeq run are trimmed and aligned to reference sequence. Wildtype, unmodified homology-directed repair (HDR) and nonhomologous end-joining (NHEJ) events from representative blastocysts were binned. All embryos were mosaic showing various combination of editing events. **(F)** The results from collating replicates over several weeks and showing editing events were shown (120 blastocysts total: 43 symmetrical, 24 asymmetrical, and 53 reverse-assymmetrical). Percentage of embryos showing no HDR reads, <10% HDR reads (low HDR frequency), and >10% reads are shown. Asymmetric oligo was least efficient among the three oligos tested. **(G)** Percentage of embryos showing one-third of gene targeted alleles (>33 % HDR); and **(H)** greater than half of the modified alleles (>50 % HDR) are shown. Reverse-asymmetric oligos resulted in better targeting efficiencies with greater number of embryos showing high HDR frequency. FWD, forward; REV, reverse.

In order to generate G127V variant, “GGA” sequence coding for glycine at position 127 (nucleotide position 381) was targeted for conversion to “GTA” to code for valine. Conversion of GGA to GTA creates an AccI restriction enzyme site (*GTATAC*) in the modified allele to allow for restriction fragment length polymorphism (RFLP)–based screening for gene targeting efficiencies. Two sgRNAs with an “NGG” PAM motif, one near the target site on the sense (forward [F]) strand and another overlapping the target site on the antisense (reverse [R]strand ) were shown (Table 1; Fig. 1A). In initial trials, precomplexed CRISPR RNPs with corresponding F- or R- sgRNA were tested alongside a symmetric 200 bp single-stranded oligodeoxynucleotide (oligos) with the targeted “TA” sequence in the middle (200 bp; 99 bp TA 99 bp; Table 1). Injected embryos were allowed to develop to blastocyst stage, at which time the blastocysts were lysed in a blastocyst lysis buffer (50 mM KCl, 1.5 mM MgCl_2_, 10 mM Tris pH 8.0, 0.5% NP-40, 0.5% Tween-20, and 100 μg/ mL proteinase K), PCR amplified, and restriction digested with AccI restriction enzyme and resolved on a 2% agarose gel. Successful targeting was assessed by resolution of the wildtype and two AccI generated fragments (526 bp wildtype and 225 and 301 bp long) on the gel.

#### Experiment 2. Design and validation of targeting reagents (sense versus antisense oligos)

Following the validation that R-guide was effective in engineering targeted modifications, all subsequent experiments for reagent optimization and for generation of live animals below was performed with the same guide. Two symmetrical oligos (200 bp; 99bp TA 99 bp and 99 bp AT 99 bp), one targeting the sense strand and another the antisense strand, were injected into the presumptive zygotes, and the blastocysts at 8 days of culture were screened for desired HDR outcomes using RFLP approach. Successful targeting was assessed by resolution of two fragments (amplicon size 641 bp; digested products 313 bp and 328 bp long) on a 2% agarose gel. Following the initial validation of the reverse symmetric oligo yielded better targeting efficiencies, two additional oligos targeting the reverse strand, asymmetric (96 bp AT 29 bp) and reverse asymmetric oligo (33 bp AT 92 bp) variants were designed and tested for successful gene targeting in bovine embryos using the methodology described above.

### Embryo injections, Estrus synchronization and Embryo Transfer

Commercially available oocytes were in vitro fertilized and injected with 0.2 μM of Cas9 protein with 0.2 μM of sgRNA and 1 μM targeting oligo and cultured in low oxygen environment. On day 7 after microinjection at the blastocyst stage, embryo transfer was performed nonsurgically. Twelve Holstein heifers were used as recipient animals (nine recipients for microinjected embryos and three recipients were used for noninjected control embryo transfer). Recipient heifers were synchronized by a modified controlled internal drug release (CIDR) protocol. Briefly, on day 0 a single shot of 10 μg gonadotropin-releasing hormone (buserelin acetate) was administered I/M and CIDR (1.38 g of progesterone in the silastic coil; Zoetis, NJ) inserted intravaginally. On day 7, CIDR was removed, and a single shot of 25 mg prostaglandin F2α (dinoprost tromethamine) was administered via I/M injection. Six to eight days after the onset of synchronized estrus, each recipient heifer was transferred with two blastocysts by depositing the embryos post-cervix intravaginally. Pregnancy was detected by BioPryn ELISA test (BioPryn; Moscow, ID) at 28 days and final confirmation by ultrasonography at 90 days of gestation.

### Genotyping of blastocysts and edited animals

Injected IVF embryo–derived blastocysts cultured for 8 days were washed three times with PBS-PVA (pH 7.4) medium and transferred into 9 μL of blastocyst lysis buffer and incubated for 1 hour at 65°C. The digestion was terminated by heating the mixture at 95°C for 10 minutes, and 2 μL of supernatant was used as a PCR template. Genomic DNA from tissue biopsies (ear notch and blood) from live calves were extracted using PureLink genomic DNA kit (Life Technologies, CA) according to the manufacturer instructions. Semen from 6-month-old bull calves was obtained by electroejaculation. The semen was washed twice in 1 × PBS, and the genomic DNA from the sperm was isolated by PureLink genomic DNA kit. The quantity and quality of extracted DNA were checked by Nanodrop spectrophotometer (Thermo Fisher Scientific). Genomic DNA from embryos above or extracted genomic DNA from blood and tissues were amplified by PCR, and efficiency of gene targeting was evaluated by restriction enzyme digestion (AccI). Additionally, the DNA was used for targeted amplicon sequencing on Illumina iSeq or miSeq platforms (Illumina, CA).

### Library preparation, MiSeq, and iSeq sequencing

#### MiSeq

Initial PCR was performed with 2 μL of blastocyst lysate using LongAmp Taq DNA polymerase kit (New England Biolabs). The PCR was performed using the following conditions: 94°C for 1 minute, 40 cycles of 94°C for 30 seconds, 60°C for 30 seconds, and 72°C for 1 minute. The cycling finished at 72°C for 2 minutes and held at 4°C. This was followed by a Step 1 and Step 2 PCR with Truseq PCR indexes (Illumina). After amplification, each sample was combined using an equal volume into one pool, cleaned with AMPure XP SPRI beads (ABM, Canada), quantified using Qubit High Sensitivity (Thermo Fisher), and was diluted to 4 nM. Normalized library (5 μL) was denatured and diluted to 10 pM, with a 20% 10 pM PhiX spike-in to ensure library diversity. Six hundred microliters of the library was loaded into a thawed MiSeq V2-300 cycle cartridge and sequenced using MiSeq sequencer with a Micro reagent kit to generate Fastq files.

#### iSeq run

Genomic DNA from somatic cells and sperm was isolated as discussed above, and 250 bp amplicons were generated by PCR. Adapters and unique indices were added to each amplicon using NEBNext Ultra II DNA Library Prep Kit (New England Biolabs) according to manufacturers' protocol. Briefly, PCR amplicons were purified with Clean NA NGS SPRI beads (Bulldog Bio), dA tailed, and ligated to universal adaptor sequences. Unique indices (NEBNext MuLtiplex oligos for Illumina) were added to each adapted amplicon by brief thermocycling using NEBNext Ultra II Q5 Master Mix and index/universal primer under the following conditions: initial denaturation 98°C for 30 seconds followed by 8 cycles of 98°C for 10 seconds and 65°C for 75 seconds, with a final extension at 65°C for 5 minutes. The indexed PCR products were purified with Clean NA NGS SPRI beads, and 1 μL of each indexed amplicon was quantified using a Qubit High Sensitivity fluorimeter (Thermo Fisher Scientific), then diluted to 2 nm. Then 2.5 μL of each indexed amplicon was combined to make the library along with 1 nM PhiX sequencing control V3 (Illumina) to ensure library diversity. Approximately, 20 μL of library and PhiX mixture was loaded into an iSeq 100 instrument (Illumina) for sequencing to generate Fastq files. Fastq files were aligned to the reference sequence by CRISPResso2.0^39^ to determine editing outcomes at the locus.

### Statistical analysis

Statistical analyses were performed with GraphPad Prism, version 8 software. Statistical comparisons of means were made using the unpaired two-tailed Student's *t*-test; *P* < 0.05 was considered statistically significant.

### Data availability

All sequencing files were deposited on National Center for Biotechnology Information Sequence Read Archive under accession number PRJNA641429.

## Results

### Optimization of CRISPR-Cas gene targeting reagents

An in vitro assay was used to identify optimal sgRNA and targeting single-stranded oligonucleotide (oligos). As shown in Figure 1A, two guide RNAs targeting the sense strand (F-guide) and the antisense strand (R-guide) near the target site were designed and tested. The goal was to alter residues coding for glycine to valine (GGC to GTA) at the target site as highlighted in the yellow box (Fig. 1A). Successful targeting and sequence conversion will result in the generation of a new AccI restriction enzyme site (Fig. 1A). Commercially sourced sgRNA and Cas9 protein were precomplexed and injected along with a symmetrical sense targeting oligo into IVF zygotes (Table 1). As shown in Figure 1B and C, the F-guide was not effective in introducing genetic modification at the site as revealed by restriction fragment length polymorphism (RFLP) analysis with AccI (Fig. 1B and C). Therefore, all subsequent experiments were performed with R-guide. Following initial evidence of an active R-guide, two different 200 bp symmetrical oligos targeting the sense (99 bp TA 99 bp) and antisense (99 bp AT 99 bp) strand on the *PRNP* locus were similarly tested. In these trials with the R-guide, antisense oligos had better targeting efficiencies compared to the sense oligos (Fig. 1D).

A final variation of the reagents tested was a refinement of the antisense targeting oligo. Three antisense oligos with unique alignments were tested for gene targeting frequency. These include symmetric (identical length target site flanking sequences; 99 bp AT 99bp), asymmetric (longer 5′ flanking sequence; 96 bp AT 29 bp), and reverse-symmetric antisense oligos (longer 3′ flanking sequence 33 bp AT 92 bp) (Table 1). To evaluate editing outcomes, targeted amplicon sequencing of 120 blastocysts (from multiple rounds of injection with the three targeting oligos) was performed using Illumina MiSeq platform (Fig. 1E). A minimum of 3000 reads were obtained per blastocyst (eight days after zygote injection) providing a >10 × coverage across the target site. The reads obtained were aligned to the reference sequence and collated as either HDR, NHEJ, unmodified/wildtype or “other modifications events.” Similar to preliminary experiments, all injected embryos showed a range of genetic modifications, from no modifications (unedited) to low frequency HDR with <10% of HDR reads (referred as <10% HDR) modifications, and a few with high frequency HDR reads (>50% HDR). For generating live HDR edited calves, a targeting oligo that yields one-third to one-half of the alleles with HDR modification (>33% or >50% HDR reads) is desirable. Among the three treatment groups, reverse-asymmetric oligo yielded better targeting efficiencies and HDR outcomes, whereas asymmetric oligo performed poorly (Fig. 1F–H). These in vitro optimization experiments identified R-guide, and reverse-asymmetric oligo as ideal candidates for editing this locus.

### Embryo injection and embryo transfer to generate edited calves

Precomplexed CRISPR RNPs (R-guide) and reverse asymmetric antisense oligo were injected into IVF-derived zygotes and transferred into synchronized heifers at blastocyst stage to generate *PRNP* edited animals. Noninjected embryos served as controls for embryo transfer and for evaluation of embryo culture and embryo transfer technique. A summary of pregnancy outcomes from embryo transfers was shown in Table 2. Six noninjected control blastocysts transferred into 3 recipients (2 blastocysts/recipient) resulted in one pregnancy (16% efficiency) that went to term and resulted in the birth of a live calf. Another 18 microinjected blastocysts were transferred into nine recipients. Six of the nine recipients went to term and delivered seven live offspring (44% efficiency) and one stillborn calf (two twin pregnancies; Table 2). Of the seven live calves, one calf was a heifer, and the remaining six were bull calves, biasing the ratio toward male offspring.

**Table 2.** **Pregnancy outcomes following embryo transfer**

Recipients	Treatment	Animal ID	Pregnancy outcome	Calving data	Sex of offspring
1	bPRNP	752	Pregnant	Twins (1 normal +1 stillborn)	Male^*^
					Female^SB^
2	bPRNP	769	Pregnant	1 calf	Male
3	bPRNP	772	Nonpregnant		
4	bPRNP	774	Pregnant	1 calf	Female
5	bPRNP	786	Pregnant	1 calf	Male
6	bPRNP	789	Pregnant	2 calves (1 euthanized)	Male
7	bPRNP	804	Nonpregnant		
8	bPRNP	819	Nonpregnant		
9	bPRNP	827	Pregnant	1 calf	Male
10	Control	806	Nonpregnant		
11	Control	809	Pregnant	1 calf	Male
12	Control	829	Nonpregnant		
			Total live calves at birth	6 edited calves;	
				1 wildtype control calf	

^*^ Calf 752 male died two days after birth.

bPRNP, bovine prion protein.

### Genotypic evaluation of edited calves

**FIG. 2.**
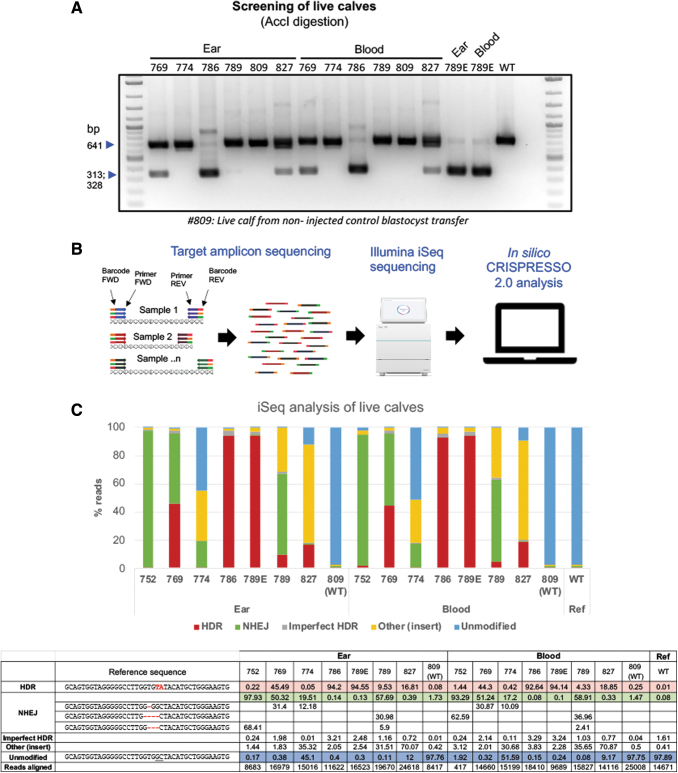
Genotyping of edited calves. **(A)** Ear and blood biopsies from calves were utilized for DNA isolation and for genotyping. The PCR amplicons were purified, digested with AccI and resolved on an agarose gel to identify successful gene targeting outcomes. **(B)** High throughput targeted amplicon sequencing on the iSeq platform. FastQ sequence output files were analyzed using the CRISPResso 2.0 platform. **(C)** % of HDR, NHEJ, unmodified, and other events (insertions, transpositions, other modifications) were binned and shown in the graph. All offspring were edited and are mosaic and have a combination of HDR, NHEJ, and other events. Calves 752 and 774 had low HDR events. Calves 769, 786, and 789 have high HDR frequencies. Calves 769 and 789 have a high frequency of 1 bp and 4 bp out-of-frame mutations.

**FIG. 3.**
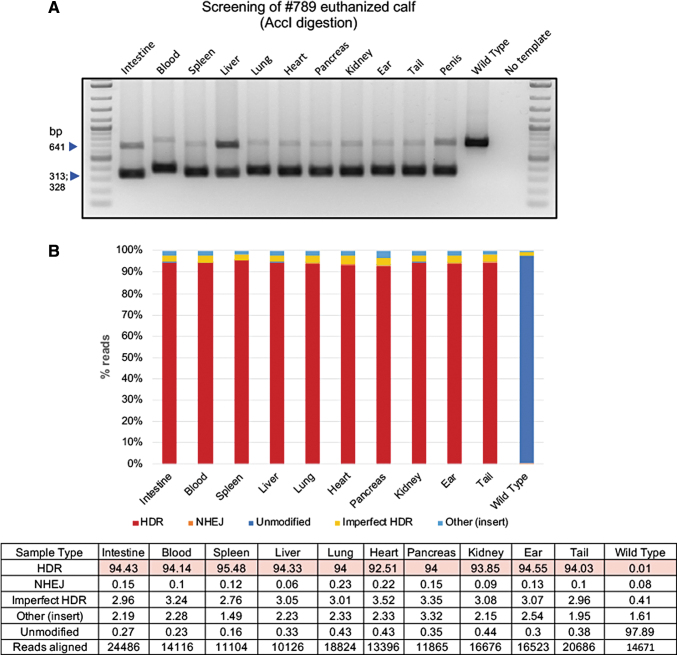
Genotyping of several tissues from a live calf with high HDR frequencies. **(A)** Biopsies from several tissues in addition to ear and blood, including intestine, spleen, liver, lung, heart, pancreas, kidney, and tail were harvested and DNA isolated for genotyping. The PCR amplicons were purified, digested with AccI and resolved on agarose gel to identify successful gene targeting outcomes. **(B)** High throughput targeted amplicon sequencing on the iSeq platform. FastQ sequence output files were analyzed using the CRISPResso 2.0 platform. % of HDR, NHEJ, unmodified and other events (insertions, transpositions, other events) were binned and shown in the graph. Results from all tissues identified events within a smaller range, highlighting the homogeneity of editing across all tissues.

**FIG. 4.**
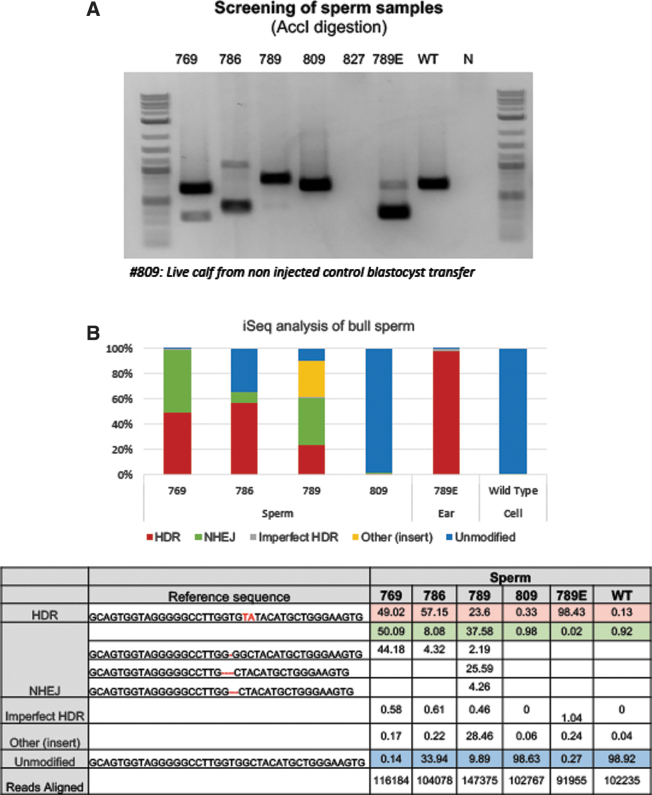
Genotyping of spermatozoa from a live calf with high HDR frequencies. **(A)** Genomic DNA from spermatozoa was isolated for genotyping. The PCR amplicons were purified, digested with AccI, and resolved on agarose gel to identify successful gene targeting outcomes. Genomic DNA from euthanized calf with high HDR edits (789 E) and wild type cells were used as controls. We could not recover semen from one of the electro-ejaculated bull calf (827) and was not incorporated in the iSeq run. **(B)** The % of HDR, NHEJ, unmodified and other events (insertions, transpositions, other events) were binned and shown in the graph.

To genotype the calves, genomic DNA was extracted from ear notch and blood of all offspring. PCR amplification across the target site was performed using a low throughput RFLP analysis (Fig. 2A) as well as a high throughput Illumina iSeq platform (Fig. 2B). A range of 8,417–25,008 sequence reads from the output FastQ files per each animal were analyzed using CRISPResso 2.0 software (with an exception of one sample with low reads: #752 blood). As shown in Figure 2C, all calves from injected embryos were edited. As expected from in vitro screens, all calves were mosaics and had a varied combination of NHEJ- and HDR-mediated repair outcomes. Among the edited calves, HDR-mediated introduction of G127V variant was identified in five of six calves at frequencies ranging from 9.53% to 94% from ear, and 4% to 93% from blood (Fig. 2C) samples, with three of the six live calves showing >33% HDR reads (numbers 769, 786, and 789E), and two calves with >90% HDR reads ( 786 and 789E). Two of the six calves (769 and 789) had a greater representation of 1-bp and 4-bp out-of-frame missense NHEJ events resulting in *PRNP* null genotype. We euthanized one of the founders (789E), which developed health problems unrelated to *PRNP* editing, and collected samples from eight different tissues (in addition to ear and blood sample), isolated DNA and performed genotyping in these different tissues via a similar low throughput RFLP (Fig. 3A) and high throughput iSeq analysis (Fig. 3B) to identify whether the genotypes from ear and blood accurately capture the allele frequency within the entire animal. As shown in Figure 3B, alignment of reads from various tissues were within the range of frequencies identified from ear and blood samples showing similar high HDR frequencies (∼94%). To confirm germline transmission of edited alleles, semen from five bull calves at 6 months of age were electro-ejaculated. With the exception of one bull calf, spermatozoa was obtained from the ejaculate (Fig. 4A). iSeq analysis of spermatozoa identified frequencies of HDR edited allele ranging from 23% to 57% among the three edited calves (Fig. 4B).

In summary, from one round of embryo transfers we have generated three founders with high-frequency of germline transmission of G127V alleles (calf number 769, 49.02%; calf 786, 57.15%; and calf 789: 23.06%; sperm), with one of the founders also carrying high frequency of 1-bp deletion *PRNP* allele (769, 44.18% reads).

## Discussion

The major goal of this manuscript was to establish a gene targeting reagent optimization pipeline to generate high frequency HDR modifications in embryos, so that the findings can be readily translated to generating live edited animals. As evidenced in the manuscript, two major competing forces need to be balanced for generating genome edited cattle for eventual commercial applications—the first being high efficiencies of genome editing and the other being pregnancy outcomes. The later which is dependent on the quality of the embryos, is also influenced by choice of editing reagents. In a nutshell, optimizing genome editing reagents is a necessary first step for improving editing outcomes and for generating high quality embryos for successful pregnancies. As we have observed and similarly noted by others in the field, not all CRISPR sgRNAs and targeting reagents are equal in their targeting efficiency. For example, the F-guide with a CRISPR cut-site farthest (5 bp) from the targeted site resulted in no discernible gene targeting compared to the R-guide with a cut-site that was 2 bp from the target site and embedded within the spacer sequence; similarly, targeting oligo cis- to cut-site (antisense oligo) resulted in higher efficiencies than the sense oligo. In a further refinement of this screening process, we adopted a high throughput Illumina miSeq platform to perform targeted amplification at 10 × coverage across the cut-site. This high throughput screen presents a quantitative rather than a qualitative readout and provides an unbiased assessment of various genome editing outcomes. This is the first manuscript, to our knowledge, that systematically investigated the use of CRISPR-Cas system for HDR-mediated gene targeting directly in embryos for generating live gene targeted calves.

Following the *ex vivo* optimization of reagents that resulted in optimal targeting efficiencies, we have proceeded with embryo injections and performed nonsurgical embryo transfers. All six resultant offspring were gene edited confirming high efficiency of editing following CRISPR RNP injections, consistent with our optimization experiments. Likewise, as reported by several groups, mosaicism in the injected embryos was a consistent outcome.^40^ Mosaicism is especially a major concern for genome editing in cattle with long pregnancy and generation intervals. Recent publications have attempted to overcome this bottleneck, including modifying the CRISPR reagents and timing of the injections.^41,42^ This will remain to be tested in livestock and will be a focus of our future investigations. Another interesting observation was inconsistency in the representation of edited alleles between soma (tissues) and germline (sperm). Although, we did not identify greater variation among the frequencies of edited alleles among various somatic tissues analyzed (Fig. 3), the same was not the case for germline (Fig. 4). This could be because, the primordial germ cells—the precursors for sperm and eggs—emerge from a small cluster of cells in the primitive streak stage gastrulating embryo and likely represent edits within a smaller cohort of cells. Regardless, from a round of embryo transfers we have generated five edited animals; two of the founders have high HDR frequencies (calves 769 and 786) and will be used for mating and propagation of the HDR edits. Likewise, two calves have high NHEJ efficiencies with either a one nucleotide (769) or a four-nucleotide deletion (789) that result in out-of-frame deletions, will be similarly utilized for generating *PRNP* null calves. These will be part of the future efforts and are beyond the scope of current publication. In conclusion, we have established a pipeline for reagent optimization, high throughput screening, and embryo transfer to generate gene targeted live cattle via zygotic injections.
